# The Electric Illumination of the Bladder and Urethra

**Published:** 1888-09

**Authors:** 


*The Electric Illumination of the Bladder and Urethra.* By E. HURRY FENWICK, F.R.C.S. Pp. 176. London: J. & A. CHURCHILL. 1888.

Mr. Fenwick has done surgeons a real service by making a special study of electric endoscopy as applied to the diagnosis of diseases of the bladder and urethra, and by so promptly and efficiently placing the results of his experience at their disposal. The work before us is well and clearly written, is sufficiently illustrated, and gives exactly the amount of information on the subject that is wanted. That there is a future before electric endoscopy in the treatment of disease, there can be no doubt: the history of future improvements must date from the publication of this work.

